# Prevention and preparedness for missing episodes in the care of persons living with dementia in Sweden – a document study

**DOI:** 10.1186/s12913-026-15019-7

**Published:** 2026-06-26

**Authors:** Rebecca Stenberg

**Affiliations:** Department of Management and Engineering, Linkoping University, Linkoping, Sweden

**Keywords:** Dementia care, Missing person, Prevention, Preparedness, Risk assessment

## Abstract

**Background:**

People living with dementia are more vulnerable, which increases the risk of harm during a missing episode. Although national guidelines exist for dementia care there are no specific standards for preventing, managing, or following up on missing episodes among people living with dementia in care settings. This study aimed to describe and analyse current local documents and checklists used in elderly care and home care services in Swedish municipalities, focusing on prevention and preparedness for missing episodes, and to identify key areas for improvement.

**Methods:**

A multiple document case study was conducted using materials from one county council and eight municipalities, including care homes and home care services, examining instructions, checklists, and routines for situations where a person living with dementia is at risk of going missing or has gone missing, one or several times.

**Results:**

Findings revealed substantial gaps in all participants documents, particularly regarding the updates of the documents, lack of preventive measures except technical solutions, delays of police notification due to inadequate instructions in case of a missing incident, limited understanding of risks and of police procedures as well as insufficient collaboration, Moreover, the decision to contact the police could be delayed for several hours and, in some cases, even days. Neither the resident nor their next of kin were actively involved in any preventive activities or risk assessments.

**Conclusions:**

Local guidelines and checklists in Swedish elderly- and home care services demonstrated substantial deficiencies in the prevention and preparedness of missing episodes involving persons living with dementia. Fundamental elements of preparedness were also lacking, including knowledge‑based collaboration with the police. Taken together, the findings suggest that admission to elderly care with exclusive emphasis on voluntariness and freedom does not necessarily increase safety for persons living with dementia. Safety and person‑centred care should be understood as mutually reinforcing rather than competing objectives.

**Clinical trial number:**

Not applicable.

**Supplementary Information:**

The online version contains supplementary material available at 10.1186/s12913-026-15019-7.

## Background

In Sweden, as in many other countries, the number of people living with cognitive impairment or dementia is steadily increasing [1, 2], while health and social care services face growing challenges due to limited resources and staff shortages [3, 4]. Dementia is an umbrella term encompassing several diagnoses, and in this article the terms “dementia” [4–6] and “persons living with dementia” are used in line with international practice [3, 7].

Persons living with dementia, including Alzheimer’s disease, frequently exhibit and experience impairments in orientation and navigation [6, 8, 9], increasing the risk of becoming lost [1, 5]. Communication difficulties may further prevent individuals from seeking help [1, 10]. As a result, missing episodes are common [3, 11, 12]; approximately, although not validated, it is reported that six out of ten individuals with dementia experience at least one such episode, which substantially increases the risk of harm [1, 3, 5].

While many persons living with dementia remain in their own homes with support from relatives or home care services, others reside in care homes to ensure adequate care and reduce risks [13]. Although most disappearances occur from private homes, missing episodes also take place in residential care settings [3]. In Sweden, dementia care is integrated into the care of old people [14]. Home care and residential services are provided by municipalities or private actors, while regional healthcare services are responsible for diagnostics and clinical care. Responsibility for preventing disappearances and preparedness for missing incidents therefore rests with municipal or regional authorities and, in some cases, with individual care providers who have a legal duty of care. In this study, the term “elderly care”, frequently employed in international and comparative contexts, is therefore used to denote a range of care arrangements, of which all have some kind of responsibility for prevention and preparedness for missing episodes.

Several countries, including Sweden, have aligned national dementia care guidelines with the World Health Organization’s (WHO) Global Action Plan on Dementia [2]. These guidelines emphasise high‑quality care combined with the greatest possible freedom regarding living conditions and mobility [11, 14, 15]. Dementia care should promote dignity, autonomy, social participation, and freedom of movement. Swedish legislation explicitly prohibits the confinement of persons living with dementia [14]. However, guidelines provide limited guidance on how to safeguard individuals despite their heightened vulnerability, creating tension between person‑centred care principles and the need to ensure safety. This tension is particularly concerning given the evidence suggesting that only about 50% of persons living with dementia survive more than 24 h after going missing [16, 17]. Similar challenges have been documented across several Western countries [3, 9].

Previous research highlights significant gaps in preventive strategies, such as insufficient measures to mitigate the risk of missing episodes. In addition, when missing episodes do occur, in preparedness: the implementation of planned, trained, and standardized response procedures aimed at ensuring rapid and effective location and rescue of the individual. Woolford et al. reported a lack of standards and routines for managing unexplained absences from care homes in Australia [9]. Aud found that preventive measures were often inadequate even when individuals had a history of previous disappearances, citing deficiencies in routines, communication, and technology such as malfunctioning alarms [4]. Other studies identify gaps in staff knowledge, architectural and environmental shortcomings, and infrastructural and societal planning failures that contribute to disorientation [12, 18]. Importantly, even when best practices are known, they are not always implemented due to staff shortages [12].

Internationally, persons living with dementia constitute one of the largest groups reported missing [1]. Repeated disappearances are common: more than 40% of missing persons with dementia have gone missing three or more times [3, 5, 11]. In Sweden, over 80% of police searches involving persons with dementia concern individuals previously reported missing [3]. Swedish police statistics indicate approximately 2,400 missing person reports involving dementia annually; an estimated 28% would not have survived without prioritised police search operations, and around 9% are found deceased [18]. Recent Swedish data suggest that nearly 40% of persons living with dementia in care settings have gone missing at least once, with almost 40% experiencing repeated incidents; close to 60% of police reports are initiated by care staff [3].

In many countries, police are responsible for responding to missing person reports [19]. In Sweden, once reported, these cases are prioritized by the police, due to their vulnerability [20, 21]. Effective police searches are based on access to trained search personnel and systematic search strategies [22]. The success is despite that dependent on preparedness within care services, including prompt reporting, and detailed information about the missing person [18, 23–25]. Preparedness could also include support for the 2–12 persons found in earlier research [26] affected by the disappearance. This includes family, friends and neighbours but also care personnel. Preventive strategies may also include structured return interviews to reduce recurrence [27], yet it remains unclear whether care services have the resources and competencies required to implement such measures.

Overall, knowledge regarding prevention, preparedness, and management of repeated disappearances among persons living with dementia remains limited, both in Sweden and internationally [12, 18]. In particular, there is a lack of empirical research examining how care providers balance autonomy, dignity, and safety within everyday care practices under resource constraints. This highlights a clear need for research focusing on care providers’ routines for prevention and preparedness in relation to missing episodes among persons living with dementia.

## Methods

The objectives of this study were:

- *To describe current local guidelines and checklists addressing prevention and preparedness for missing episodes among individuals living with dementia in Swedish elderly care and home care services.*
- *To analyse these guidelines and checklists and assess areas requiring improvement; and*
- *To identify key components for effective prevention and preparedness, thereby contributing to improved work environments for elderly care staff.*

### Study design

A multiple-document qualitative case study was conducted using documentation from eight municipal care homes and home care services, as well as one county council, focusing on safety management within these settings. These included checklists detailing procedures to follow when a patient or client goes missing, whether once or repeatedly.

Ten municipalities, one healthcare region, and two private firms across Sweden were selected by qualitative contrasting [28] to represent diverse geographical and demographic contexts, such as North Sweden/South Sweden, urban or rural areas, large or small municipality/region. They were approached by letter with a description of the project and asked to provide relevant documentation about safety and security routines and additional checklists. Seven municipalities and one region submitted documents concerning the prevention of missing episodes and checklists or instructions outlining actions to take when a resident or home care user disappears. One municipality reported having no such documents at all, and the two firms did not respond. All the responding actors, including the municipality with no documents, were included while the two firms who did not responded were excluded.

#### Cases

The eight municipalities ranged from a small rural municipality with approximately 13,000 residents to an urban city with 143,000 inhabitants. The region comprised 177,000 citizens. The municipal documents differed in both volume and age. The two oldest checklists were more than ten years old. Along with the region, they possessed documents or checklists containing 6–14 bullet points outlining procedures when a resident goes missing, until the individual is located. In four cases, these also included follow-up measures and post-incident information. Six of the documents had been revised recently, while three were more than ten years old. The documents/checklists addressed both home care service staff and staff in nursing homes, and in the case of the region, healthcare staff.

Both the municipalities and the region is anonymised.

### Data analysis

The collected documents were first systematically organised in a table of documented routines, instructions, and checklists guiding staff on what to do when patients living with dementia are at risk of going missing or have gone missing.

The information from all the actors was sorted into classes encompassing the following kinds of data as presented in Table 1.

**Table 1:** Data sorted into classes

1. Information concerning organisational structure, geographical location, and population characteristics. 2. Document details, including the documents and checklists’ revision dates, length, and records of search or missing‑person episodes, as well as characteristics of the missing individual. 3. Instructions addressing preventive measures and secondary prevention strategies.
4. Instructions regarding internal search protocols and communication with supervisors. 5. Instructions concerning interdepartmental contacts, emergency healthcare, police contacts, and communication with the missing person’s relatives. 6. Instructions outlining procedures upon the person’s return, follow‑up actions, and allocation of responsibilities.

The full comparative table over documents for prevention and preparedness for missing episodes from the eight municipalities and one region over prevention and preparedness for missing episodes from eight municipalities and one region is provided in Supplementary Table 1: *The full comparative checklist table over prevention and preparedness for missing episodes from eight municipalities and one region*.

The analysis employed within this structure thereafter directed content analysis [29] in iterative steps. A guide was constructed based on previous research regarding prevention and management of missing episodes [3, 5, 18] and studies in preconditions for efficient policework regarding search for missing persons living with dementia [18, 21, 24, 25, 30]. The result was categorised after the content as shown in Table 2.

**Table 2:** Guide for the content analysis

1. What type of document is it? Does it constitute an extended plan with routines and checklists? 2. How old is the document, and when was its most recent revision? 3. Does it include measures for prevention or secondary prevention (to prevent repeated disappearances)? 4. What does the checklist or instruction specify? Who is mentioned in the checklist, and in what order? 5. Are staff instructed to search independently, collaboratively with other staff, or with external actors (besides relatives and the police)? 6. Are relatives mentioned or contacted? For prevention? When the person is missing? At what stage in the process? Is any follow-up with relatives indicated? 7. Does the document mention contact with a manager or multiple managers? What is the manager’s role?
8. Does the document refer to contact with emergency or healthcare services? 9. Who is responsible for contacting the police, and when? Is any interaction or coordination of actions described? 10. Who is identified as responsible for searching? Are legal responsibilities addressed? 11. Are there instructions for providing a description of the missing person? What does this include—photographs? 12. How is the episode documented (for future reference or learning)? 13. Who is responsible for what when the person is found? Who should be informed? What if the person is found deceased? 14. What kind of follow-up is mentioned, and for whom? 15. Does the document address responsibility or culpability?

## Findings

The analysis of the documents used the following categories: prevention; actions taken when someone is missing; internal routines and staff assignments; contact with supervisors and managers, healthcare services, relatives, and the police; and follow‑up activities. The municipalities are referred to by letters; however, Municipality A had no documented routines or checklists available for analysis, which in itself constitutes an important finding. The age of the documents varied considerably. The two oldest were more than ten years old.

All services were governed by the Swedish Social Services Act [31], the Act concerning Support and Service for Persons with Certain Functional Impairments (LSS), (SFS 1993:387) [32], and the Health and Medical Services Act (HSL) [33],. The Policies and all operations were conducted in accordance with directives from the National Board of Health and Welfare [14], which also define the national policy for dementia care. Therefore, policy documents were similar, primarily emphasised the importance of voluntariness and the avoidance of coercive measures. Only one municipality © highlighted the importance of knowledge regarding missing incidents and the critical role of time in such situations.

### Prevention

In three cases (B, C, and E), prevention was discussed or mentioned in the checklists or documents, and in three cases (C, E, and G), secondary prevention aimed at avoiding recurrence was also noted. Preventive measures included, in two cases (B and C), the use of GPS watches or devices attached to clothing; in one municipality, a complex door‑locking system was also employed. In the fifth municipality (E), it was emphasised that residents and relatives share responsibility for preventing missing episodes: “Every resident has a responsibility based on their ability. Even if all necessary staff are present, it is impossible to always be at hand.”

Secondary prevention was implemented through dialogue with the resident in one case (G), and through support for both the resident and others affected, such as staff and relatives, in another (E). In the third municipality, a missing episode led to a risk analysis and the development of an action plan (C).

None of the examined checklists or documents referred to systematic interactive preventive activities undertaken in collaboration with the resident, such as co‑designed care planning [17]. Two municipalities referred to environmental interventions, including the use of GPS devices and locked door systems. However, these measures were not discussed with residents or relatives.

Prevention could include initial interviews concerning personal history and everyday preferences. A risk assessment for going missing could be conducted upon admission to a care home; however, this was identified in only one checklist and then as a post‑incident measure intended to prevent recurrence.

Residents in care homes cannot be responsible for preventing themselves from becoming lost or missing. Care homes remain responsible for residents’ safety in accordance with the Social Services Act [30]. The emphasis in one municipality’s documentation on residents and their relatives bearing responsibility for avoiding becoming lost is therefore questionable.

### When a person is missing

In the municipalities (B, C, D, E, F, G, and H) and at the regional level, the checklists outlined actions to be taken when a resident was not found where he or she was expected to be. In one municipality’s checklist for home‑care services (D), staff were instructed to return after two hours if the door was not opened. If the resident did not respond a second time, staff were to gather all possible information about the person’s habits, contact relatives, and inform the supervisor, who would then decide whether to alert the police.

Another municipality (C) distinguished between a person who had disappeared and a person who was missing but whose whereabouts were known. If the person had disappeared, a rapid check of the nearby environment was conducted. In other checklists (B, D, E, F, G, and H) and at the regional level, staff were instructed to begin searching themselves and to alert the GPS dispatcher, department nurse, supervisor, and/or department or area manager upon discovering that a resident was missing.

When a person was found to be missing from their residence, ward, or private home, and staff in one municipality’s home‑help service were instructed to wait for two hours before returning, it presents a significant safety dilemma. Although residents are free to come and go in accordance with national guidelines [14], a delay becomes critical if the absence is involuntary. Given that only approximately 50% of missing persons living with dementia are found alive after 24 h [6, 16, 17], a delay of several hours is highly consequential.

Additional delays arose from instructions in most municipalities and at the regional level requiring staff to search a designated area or for a specified duration before alerting the police. Furthermore, in two municipalities the instruction to use the Swedish Police Authority’s non‑emergency service number [114 14] instead of the national emergency number could introduce further delays due to waiting times in telephone queues.

#### Search conducted by staff

All but two checklists (B, E, F, G, H, and the region) instructed staff to prioritise searching for the missing person. The regional checklist specified the search area as “the entire hospital,” while another stated “all areas within 500 metres of the person’s residence.” One municipality (F) instructed staff to search for 30 min, and another (E) for 50 min. One municipality (B) provided all residents with GPS watches; when someone went missing, the dispatch centre was to be contacted, the GPS watch located, and staff were to return the person. If the individual refused to return, the police were to assist.

In most municipalities (B, D, E, F, G, H, and the region), a series of actions were required before alerting the police: searching independently, involving staff from other departments, appointing a search leader, alerting healthcare staff, and informing supervisors and management.

### Contact with supervisors and managers

Instructions to initiate a search were followed by directives to inform immediate supervisors, who were then expected to notify department managers, who in turn were required to inform area managers in a sequential, hierarchical process that often extended into the following working day. In all but one case (C), the police were to be contacted only after supervisors and managers had been informed.

Substantial delays could therefore occur before the appropriate management level authorised police contact. Even in municipalities and at the regional level where early contact with the police was encouraged, the decision often had to pass through several organisational layers or be postponed until internal searches had been completed. This reflects a limited understanding of the importance of time and the risks associated with delays—both for the missing individual and for the conditions required for effective police search efforts. In one municipality, staff were instructed to notify a designated individual responsible for contacting the police. This person was only reachable on weekdays.

### Contact with relatives

Three municipalities (C, G, and H) instructed that relatives should be contacted at an early stage when a resident was missing, whereas three others postponed communication with relatives until the police had been notified or the missing person could not be located despite police efforts (B, E, and F). One checklist advised staff to remain calm and provide support to relatives during the search [3]. Two checklists (F and G) included follow‑up procedures involving both staff and relatives.

Only three of the checklists included instructions to contact and inform relatives at an early stage of a missing‑person incident; in three others, relatives were contacted only if the person could not be found or when the police assumed responsibility for the search. Overall, relatives were consistently excluded both from preventive efforts and from discussions regarding monitoring and follow‑up.

### Contact with the police

In one municipality (C), the police were contacted immediately if a resident had disappeared, but not if the individual was missing while their whereabouts were known. Another municipality instructed staff to contact the police at an early stage using a dedicated number or the national emergency number (G). In the region and in five municipalities (B, D, E, F, and H), staff were instructed to notify the police after 30–50 min, or later, if internal search efforts were unsuccessful. In two municipalities (B and H), staff were directed to use the police authority’s non‑emergency service number.

When the police arrived, five checklists (B, D, F, G, and the region) stated that the police would “take over” or “assume responsibility.” Four checklists (B, E, F, and G) required staff to provide a detailed description of the missing person, while one checklist (C) referred to the individual’s life‑history document. One checklist (E) instructed staff to inform the police of areas already searched. Another (C) required staff to continue searching in locations not covered by the police. One municipality (G) specified that the information provided could include a recent photograph of the missing resident, if available.

Several checklists instructed staff to leave once the police arrived and not to “bother” the police officers. This results in lost opportunities for information sharing and experiential learning. Moreover, since none of the documents or checklists mention the police being present in follow up, another opportunity for collective learning—for staff, the police, the formerly missing person, and their relatives—is also lost.

### When the person is found / follow‑up

Four checklists included instructions for actions to be taken when the person was found (C, E, F, and G). Two (C and F) mentioned medical or psychiatric follow‑up; all four required notifying all searchers that the search had ended and contacting relatives. Three checklists recommended follow‑up of the incident with staff, relatives of the formerly missing person, and with the resident him‑ or herself (C, F, and G). One checklist (C) further recommended discussing issues of guilt and responsibility with ward staff.

This highlights a relatively under‑researched area concerning work‑environment impacts following missing‑person episodes in care settings. Staff members involved with a resident who goes missing may experience anxiety and feelings of guilt, particularly if the resident is not found or is found deceased. Staff may also—rightly or wrongly—be held responsible for the incident by the missing person’s relatives. Altogether, this demonstrates the need for structured follow‑up involving all parties, including, ideally, the police.

### Language

Worth reflecting upon is that although the guidelines promote care based on freedom of choice, the language used in some of these documents reflected terminology associated with criminal incarceration in Sweden. For example, the missing person was described as having “deviated” or “absconded” from their residence and the police were provided with the person’s “characteristics” or “descriptions.” Means of prevention can be “surveillance” (authors translation from Swedish).

## Discussion

The analysis identified factors which were central and recurrent. These factors are interrelated and constitute significant safety challenges in the care of people living with dementia, while also representing key areas for improvement in order to meet the objectives of the World Health Organization’s (WHO) Global Action Plan on Dementia [2]. They are discussed in greater detail below.

### Inadequate and delayed responses to missing incidents

Research by Neubauer et al. [6] identifies time delays following the disappearance of a person living with dementia as one of the most significant challenges from a police perspective. Given the high frequency with which persons with dementia go missing and the associated elevated mortality rates [17], largely attributable to vulnerability and environmental factors such as cold weather, time becomes a critical determinant of survival [18] according to the police. Internationally this is confirmed by research; There is a time window of approximately 24 h during which the survival rate is around 50% for missing persons with dementia. Beyond this period, the likelihood of survival declines dramatically [16, 17]. The longer the delay before a search is initiated, the more difficult, resource‑intensive, and time‑consuming the search becomes [6, 18, 34]. Therefore, an essential component of prevention is the establishment, maintenance, and regular updating of preparedness plans based on risk analyses and clearly defined response procedures [35].

In the present study, several existing checklists had remained unchanged for more than ten years, suggesting either that they were perceived as sufficiently adequate or, more plausibly, that they were not actively used in practice. The latter interpretation points to shortcomings in organisational responsibility and risk‑management capacity within care settings where life‑threatening missing episodes are known and recurring risks. This interpretation is further supported by the limited relevance of the instructions contained in the documents. Consequently, individuals living with dementia who move into elderly care in order to increase safety may instead be exposed to the opposite outcome in the event of a missing episode.

Although early notifications of the police were stated in several documents, other checklist instructions entailed substantial delays in reporting. This included guidance that staff should first conduct independent searches, appoint a search coordinator, and inform the police about areas already searched. While it is reasonable for staff to conduct an initial search of the immediate surroundings, systematic searching by trained search and rescue personnel cannot be replaced by untrained staff attempting to “search the entire hospital area” [24]. Additional delays arose from requirements that decisions to report a disappearance to the police had to pass through hierarchical managerial levels—from frontline supervisors to department heads and site managers—before a report could be formally filed. In some cases, this resulted in delays of several days before police involvement was initiated, as the designated individual responsible for contacting the police was only available on weekdays. This signals a lack of motivation or understanding of the difference between everyday decision making and emergency response, as well as not understanding the seriousness of these incidents as emergencies.

### Insufficient knowledge within the care organization regarding the scope and risks associated with missing episodes

The police and the elderly care services on municipal or regional level did not share any inter‑institutional statistics on the extent of missing persons living with dementia, or deaths resulting from missing incidents [36]. One reason why this statistical follow-up was not disseminated was the presence of underdeveloped statistical systems within the police [3, 30]. Another reason is that information sharing with external stakeholders has declined both in the police and in the elderly care as a result of cutbacks associated with New Public Management strategies across many public sector organizations [18]. In the absence of shared knowledge, it is difficult to understand the overall scope of the problem beyond one’s own organization, where, according to the police, the frequency of incidents could vary considerably between different residential care facilities and services ((M. Grahn, Swedish Police Authority, personal communication, 2023).

Despite the fact that nearly 60% of reports to the police in Sweden originate from care personnel [3], the checklists reviewed indicated low risk awareness regarding the severity of danger that a missing episode poses to a person living with dementia [34], as well as insufficient recognition of the importance of safety as an aspect of quality care [2, 31].

The findings of the present study are closely aligned with international research, which similarly demonstrates a lack of preparedness for missing episodes within the organization of elderly care services [4, 9, 12]. The absence of consolidated statistics in this area constitutes an international problem [3, 37] and may contribute to persistent gaps in knowledge. Other contributing factors may include insufficient resources and staffing shortages, which represent an increasing challenge within elderly care systems [3, 4].

### Overreliance on environmental and technological solutions alone as prevention and preparedness strategies

The reviewed documents and checklists revealed limited consideration of potential preventive approaches beyond environmental interventions. To avoid the use of restraints for persons living with dementia, two overarching strategies are according to research employed in response to missing episodes: internal strategies, in which elderly care services focus on prevention [38], and external strategies, in which the police maintain preparedness for efficient responses to missing‑person cases [6, 25].

Internal strategies primarily consist of environmental interventions and various forms of surveillance- and intervention technology [38]. Reviews of such interventions—including modified flooring, complex locking systems, designated indoor paths, and GPS‑enabled watches—have demonstrated no strong evidence of effectiveness when internal strategies are implemented in isolation [38]. The use of GPS devices attached to clothing, watches, or other personal items requires someone to monitor and operate the technology. This work often falls onto either care staff, who often face significant time constraints, or relatives, who may be unfamiliar with or lack confidence in using newer technologies [18]. In both strategic approaches, successful outcomes depend on the involvement and actions of additional actors and resources, among them, the resident, relatives and staff.

### Involvement of the resident and their next of kin in prevention of missing episodes

In this study, neither residents nor their relatives were identified as stakeholders, resources, or sources of knowledge in relation to prevention in any of the examined documents, nor was the resident’s participation explicitly addressed. A review of stakeholders’ perspectives on surveillance for individuals living with dementia [39] identified a scarcity of studies examining the views of residents and relatives, focusing instead on the perspectives of care personnel. The review further emphasized the importance of involving all stakeholders in decision‑making processes, given their differing understandings of the purpose and value of surveillance as a preventive strategy.

For an internal preventive strategy to be effective both technological solutions and other preventive measures must be combined according to earlier research [38]. Participation in the prevention of missing episodes must also be based on the residents’ involvement and on an understanding that not all disappearances are irrational; rather, they may, for example, reflect dissatisfaction with the residential environment, staff, or care provision [40, 41]. Consequently, it is essential to identify and address individual risk factors and to actively involve both the residents and their relatives in care planning processes.

### Limited mutual understanding of each other’s routines and insufficient inter-agency collaboration between care staff and the police

In all but one of the reviewed checklists, the police were not to be contacted until staff had conducted their own searches, in several cases for prolonged periods, and until a decision to notify the police had been approved by the appropriate authority within the care organization. As earlier mentioned, these procedures resulted in substantial delays. Contrary to assumptions that the police should not be “bothered” unnecessarily, as well as other reasons for delaying notification, the police prefer to be informed immediately when a person living with dementia is reported missing in order to ensure optimal conditions for a successful search [18, 25]. Another persistent misconception, widely reinforced by media portrayals, is that the police will not initiate a search within the first 24 h of a missing person’s case [18].

The instructions to withdraw once the police arrived also resulted in missed opportunities for experiential learning and inter‑professional dialogue. Moreover, as none of the follow‑up documents or checklists mentioned police participation, an additional opportunity for collective learning—involving staff, the police, the formerly missing person, and their relatives—was also lost.

As a collaborative resource the police in Scotland, have introduced the Herbert Protocol, an information‑sharing document designed to improve preparedness within care services and among relatives by providing police with relevant information to guide searches for persons living with dementia [23].

In Sweden, an underutilised collaborative information resource for the police is the life‑history documentation compiled when a person enters residential or dementia care [18]. This documentation contains information required by the police to initiate a search, but it is not consistently accessible to the police or available on a 24/7 basis. This source of information is currently largely unknown by police officers.

### Preparedness

Drawing on the World Health Organization’s definition of preparedness as a planned and systematic response [42], and Kaneberg’s description of preparedness as the capacity of a system to partially and temporarily shift focus and modes of operation—such as decision‑making processes—in response to critical situations [35], it can be concluded that the documents and checklists of all participating actors required substantial improvement to serve as an adequate basis for preparedness. This applied both to preparedness for locating and rescuing missing persons living with dementia but also preparedness for other adverse events requiring coordinated responses.

### Implications for practice and policy

The findings of this study demonstrate that, due to limited knowledge about the risks associated missing episodes for persons living with dementia, and delays in response to and reporting to the police, residents may remain missing for hours and be exposed to serious threats to life before systematic searches are initiated.

At the immediate practice level, the findings indicate that suspected missing‑person incidents involving persons living with dementia should be treated as time‑critical emergencies, with time emerging as a central factor. In line with research on preparedness [18, 35], clear and regularly updated procedures are required to ensure that frontline staff or designated supervisors have the authority to contact the police without delay, rather than routing decisions through hierarchical managerial structures.

After an initial search of the immediate surroundings, staff can shift to other essential tasks, such as informing and supporting relatives. Previous research [18] also suggests that the police should be provided with a detailed and up‑to‑date description of the missing person, and that systematic post‑incident follow‑up should be implemented to support the resident, relatives, and affected staff, while also enabling organisational learning and the continuous improvement of routines.

At the organisational level, prevention and preparedness for missing episodes should be integrated into routine dementia care. Preparedness should be grounded in systematic risk analysis and result in a documented preparedness plan [35] that defines decision‑making authority and the allocation of responsibilities, and that is subject to regular review.

Preventive efforts [18] should be based on ongoing dialogue with residents and relatives, combining technical solutions with environments that are easy to navigate and supportive measures that promote residents’ willingness to remain within the care setting [38].

Admission procedures should actively involve the resident and, where possible, their relatives and, as observed in one municipality, include a structured risk assessment conducted through dialogue to support decision‑making. These procedures may also include consideration of environmental measures, such as GPS devices or other forms of monitoring. Life‑history documentation should be formally established and regularly updated [18]. Where appropriate and with informed consent, mechanisms to ensure police access to relevant information on a 24/7 basis should be explored.

Strengthened inter‑agency collaboration—through shared routines, joint training, and post‑incident dialogue—may further reduce delays and misunderstandings in emergency responses. At the policy level, the findings highlight a need to better align dementia‑care guidelines with safety and preparedness considerations. While current frameworks appropriately emphasise autonomy, voluntariness, and freedom of movement, the absence of explicit guidance on risk management and preparedness for missing‑person episodes represents a structural gap. National guidelines could be strengthened by recognising missing episodes as foreseeable, high‑risk events and by defining minimum standards for prevention, preparedness planning, and inter‑agency coordination.

These implications suggest that there are substantial challenges in reducing risk for persons living with dementia who go missing from care settings as summarized, together with suggestions for how to meet them in Table 3. They further indicate that ensuring safety should not be understood as a contradiction of, or restriction on, quality person‑centred care, but rather as an asset and a prerequisite for quality and sustainability in dementia care systems, while also strengthening system capacity to manage other forms of stress and crisis.

**Table 3:** Challenges and suggestions for how to meet them

Challenges	Suggestions
Inadequate and delayed responses to missing incidents.	Staff should be informed about the severe risks associated with missing episodes. Clear routines for immediate police notification by the nearest supervisor or manager should be established. Missing persons and Staff experiences following incidents should be systematically followed up, and routines revised accordingly.
Insufficient organizational knowledge of the scope and risks of missing episodes	Preparedness standards for missing episodes should be developed as part of quality-of-care guidelines. Inter-agency sharing of statistics at local, regional, and national levels should be established.
Overreliance on environmental and technological solutions	Admission routines could include systematic risk-assessment discussions and decisions regarding technical solutions. A documented life-history interview should be conducted, and the police should be granted access to relevant parts of the life-history documentation, subject to informed consent.
Lack of involvement of residents and next of kin	Residents and relatives could be involved in admission decisions and care planning. Regular review meetings with residents (and relatives, where appropriate) could be held and follow-up conversations conducted after missing episodes to help prevent recurrence.
Limited mutual understanding and inter‑agency collaboration with the police	Local information meetings could be arranged between care personnel and the police to improve understanding of police routines and response priorities, as well as awareness of and access to life-history documentation.
Insufficient preparedness for missing incidents and other crises	Preparedness plans based on risk analysis are required under occupational health and safety legislation and legislation governing the care of persons living with dementia. Such plans may also include response procedures for missing-person incidents and should be coordinated with the police and other relevant actors. In general, preparedness plans should be tested through training exercises and reviewed annually

## Strengths and limitations

### Strengths

This study addresses an important and under‑researched area in dementia care, where the findings have potential relevance for reducing harm among persons living with dementia and for mitigating substantial downstream demands on the police. By focusing on documented routines and checklists, the study provides insight into how preparedness for missing‑person episodes is formally articulated and governed within elderly care organisations. Although based on a limited and deliberately selected geographical sample, the analysis is well anchored in international research [3, 9, 11, 41], supporting the broader relevance of the findings.

The study clearly demonstrates gaps in preparedness, risk awareness, and inter‑agency collaboration, and highlights how delayed responses and limited coordination may increase risks to life and health. It also identifies a key tension between person‑centred care grounded in voluntariness and autonomy, in line with World Health Organization guidelines, and the need to ensure safety—suggesting that safety risks are insufficiently integrated into prevailing conceptions of quality care. An additional strength is that the proposed improvements are largely organisational [18]. in nature and can be implemented with minimal direct financial costs.

### Limitations

In line with the limitations of qualitative case studies [29], the findings of this study cannot be generalized to elder care prevention and responses to missing episodes; rather, they provide illustrative examples based on selected cases. The analysis draws on documents from one region and eight municipalities of varying sizes and geographical locations across the country, including instructions and checklists outlining how such situations should be handled. This diversity offers several avenues for further investigation and follow-up research. Future studies may include larger samples of municipalities, allowing for broader generalisation, as well as a wider range of incident types beyond missing person cases.

A further limitation is that the study is based solely on document analysis of which some were more than ten years old. The documents were not supplemented with interviews nor linked to police incident reports. This restricts the conclusions that can be drawn regarding actual practice, as documents may reflect espoused theory rather than theory-in-use [43] in a highly strained Swedish elder care system, affected by cutbacks [18] and persistent staffing shortages [3], practice may not always align with formal guidelines. To some extent this is confirmed since none of the documents mentioned Covid 19 pandemia, although it affected the elderly care [44] and four of the documents have expiring dates years 2021 or 2022 or later.

Another limitation is that the study includes only municipalities and one region, with no participation from privately operated care providers although privately operated care are known for lower staffing [44] which is connected to resident safety in general and higher risks for missing episodes [3].

Finally, the study largely adopts a policing perspective (21,29), which has informed the identification of key issues related to both prevention and preparedness.

## Implications for future research

Future research should include larger‑scale studies and mixed‑methods designs to further examine and refine how policies, instructions, and everyday practices currently operate in relation to the prevention of and preparedness for missing‑person episodes involving persons living with dementia.

In a comprehensive mixed‑methods study, residents, relatives, and staff at different organisational levels should be included through qualitative interviews. Where ethically and legally feasible, such studies could be complemented by police incident reports linked to the participating care facilities, allowing for triangulation between organisational routines, lived experiences, and documented emergency responses.

A related area in need of further investigation concerns staff experiences of missing‑person incidents from a work‑environment perspective, including emotional impact, perceived responsibility, and organisational support.

In addition, more comparative research is needed on missing‑person episodes involving persons living with dementia, particularly regarding fatalities. According to Swedish police statistics, the proportion of persons living with dementia who are found deceased following a missing episode is considerably higher in Sweden (approximately 9%) than in, for example, the United Kingdom or Scotland, where the corresponding figure is around 2.5% [5, 30]. Existing statistics do not clarify to what extent these deaths involve residents who went missing from care homes, highlighting an important gap for future research.

## Conclusions

This study analysed current local guidelines and checklists addressing the prevention, secondary prevention, and preparedness for missing episodes involving persons living with dementia in elderly care and home care services in Sweden. The analysis was based on documents collected from eight municipalities and one county council.

Regarding concrete preventive measures, the findings indicate limited risk awareness and insufficient understanding of the severity and time‑critical nature of missing episodes. Several of the reviewed documents were more than ten years old and did not reflect current empirical knowledge regarding mortality risk and search urgency. In practice, efforts to avoid “unnecessarily bothering” the police could result in delays of several hours—and in some cases days—before a missing person report was filed. These delays were reinforced by organisational routines requiring staff to conduct independent searches and by hierarchical decision‑making structures in which only designated individuals were authorised to contact the police, sometimes restricted to office hours. As a consequence, police search efforts could be delayed and impeded by misunderstandings of roles, responsibilities, and time sensitivity, as well as by inadequate inter‑agency collaboration.

In terms of preparedness, none of the analysed documents articulated a systematic preparedness plan for missing episodes, including structured risk analyses or clearly defined action plans for rapid and effective response. Nor did the documents demonstrate preparedness for other serious or crisis‑type events. Taken together, the findings suggest that admission to elderly care does not necessarily increase safety for persons living with dementia and may, in certain situations, introduce new and serious risks. An exclusive emphasis on voluntariness and freedom of movement therefore does not, in itself, guarantee high quality of care.

At the policy level, safety must be recognised as an integral component of quality in dementia care. Considerations of safety and person‑centred care should be understood as mutually reinforcing rather than competing objectives. Missing episodes should be explicitly acknowledged as a core issue within quality assurance and preparedness frameworks in dementia care.

At the organisational level, there is a clear need for systematic prevention and preparedness plans that actively involve persons living with dementia and their relatives. Preventive work should include structured life‑history interviews and clearly regulated access—where consent is obtained—to relevant information for the police on a 24/7 basis to support search efforts. Initial risk assessments should be conducted upon admission and, where appropriate, combined with environmental or technological measures. Preparedness must be grounded in an organisational understanding of the actual risk of missing episodes, based on knowledge of previous incidents, awareness of associated dangers, and recognition that such events constitute medical and social emergencies requiring immediate action within strict time limits.

## Supplementary Information

Below is the link to the electronic supplementary material.

Supplementary Material 1: Supplementary Table 1: The full comparative checklist table over prevention and preparedness for missing episodes from eight municipalities and one region

## Data Availability

All data generated or analysed during this study are included in the appendix to this published article [and its supplementary information files]. The document presents an academically revised and context-adapted version of the comparative checklist table supplied by eight Swedish municipalities and one health care region. The language has been standardised into formal British English while retaining the original meaning and intent of each entry. All the original materials are available and can be available from the corresponding author on reasonable request.

## Declarations

### Use of large language models

Language editing and translation to academic English in the paper and the table were supported by Microsoft Copilot. The tool was used solely for linguistic refinement; no substantive content was altered.
