# Supplementary material for: Prevention and preparedness for missing episodes in the care of persons living with dementia in Sweden – a document study
Source: BMC Health Serv Res. 2026 Jun 26;26:872. doi: 10.1186/s12913-026-15019-7 (PMC13312658; doi:10.1186/s12913-026-15019-7)
Supplement: Supplementary file 1 — Supplementary Material 1: Supplementary Table 1: The full comparative checklist table over prevention and preparedness for missing episodes from eight municipalities and one region [file 12913_2026_15019_MOESM1_ESM.docx]

# Supplementary *Table 1: The full comparative checklist table over prevention and preparedness for missing episodes from eight municipalities and one region*

This supplementary file contains the full comparative checklist table (eight municipalities labeled A - H and one health care region) as referenced in the manuscript. Several of them specify stepwise measures. Where indicated, these steps have been presented in the table. The wording has been standardised into academic British English, retaining the original meaning. Due to variations and gaps in the source material, some entries remain marked with a dash to indicate missing information.

| **Category** | **Municipality A** | **Municipality B** | **Region** | **Municipality C** | **Municipality D** | **Municipality E** | **Municipality F** | **Municipality G** | **Municipality H** |
| --- | --- | --- | --- | --- | --- | --- | --- | --- | --- |
| Location in Sweden | Northern Sweden | Northern Sweden | Sweden | Central Sweden | Eastern Sweden | Eastern Sweden | Southern Sweden | Southern Sweden | Southern Sweden |
| Citizens | 23,000 | 49,000 | 177,000 | 24,000 | 143,000 | 36,000 | 13,000 | 17,000 | 86,000 |
| Document revised | No document | 2023 | 2022 | 2009 | 2019 | 2011 | 2021 | 2019 | 2022 |
| Prevention | — | GPS alarm; locked door; secure door lock mechanisms | Unknown | GPS alarm when required | — | Information provided to residents and relatives with emphasis on responsibility | — | — | — |
| Secondary prevention | — | — | Unknown | Risk analysis and action plan | — | Support to affected individuals | — | Dialogue with the resident | — |
| Checklist coverage | — | Action plan (6 steps): from discovery of absence to notification of supervisor and next-day staff | From discovery of absence to locating the person (4 steps) | Policy document, routines, and checklist | Documented routines in 5 steps.  return after 2 hours if there is no response at the door | Action plan (6 steps) | Routine (13 steps) | From discovery of absence to follow-up (14 steps) | Checklist (7 steps) |
| Search by personnel | — | Alert dispatch center: staff search and engage home help services if needed | Step 1 Extended search by all staff | Step 1 rapid check of nearby environment | Step 2 Review records; Contact hospitals | Step 1–2: systematic search of premises and nearby areas; show police where searches conducted | Step 1–4: staff search up to 500 m; internal search leader appointed | Step 1  Staff search independently for 30 minutes | Step 1  Staff searches close premises |
| Contact with relatives | — | If the person is not found, relatives are notified | — | Step 3 Notify legal guardian and relatives | Step 3 After that; Contact relatives | Step 6 Relatives notified last | Step 8 Relatives notified | Step 2 relatives notified after 30 minutes and during follow-up | Step 3 relatives notified |
| Contact with manager | — | Notify head of department if the person cannot be located | Arrange search of hospital area | Step 2  Area manager decides on Police contact | Step 4  After that; Contact manager | Step 3: contact head of department, area manager notified | Step 5 Contact nurse  (Step 6) contact area manager | Step 3 | Step 4,5 Notify head of departmentand after that area manager |
| Contact emergency care | — | — | — | — | — | — | — | Step 4  Emergency care contacted | Step 2 Contact nurse |
| Contact with Police | — | Step 4  Call Police service number early | Police contacted after all internal measures exhausted | Step 4 Police always contacted when a person has disappeared (not only missing) | Step 5 Police contacted | Step 4–5 Call distress number | When emergency: call distress number; otherwise contact police | Step 5 police called after 30 minutes | Step 6 police service number 114 |
| Responsibility for search | — | Personnel until Police arrive | Police assume responsibility | Step 5  Staff search independently without disrupting Police operations | Police assume responsibility | — | Step 11  Police responsible | Police assume responsibility | — |
| Description of characteristics | — | Staff provide information on habits, medical conditions, relatives, search areas, and previous disappearances | — | Life history document used | — | Yes | Includes name, personal number, emergency contacts, characteristics, clothing, habits, previous addresses, search areas, and relatives' contact details | Police informed, Recent photos if available | — |
| Internal organisational contact | — | Deputy head of department | Chief medical officer | Maintain calm and support relatives | — | Team discussions and internal support | Step 6 Support from other home‑help units | Nurse contacted; home‑help staff assist; area manager contacted | Step 7 Assistance from other departments |
| Documentation | — | — | — | — | — | — | — | Social documentation | — |
| When person is found | — | — | Distress call cancelled | Attend to the person; contact physician for psychiatric evaluation if needed; Police may assist; relatives informed | — | — | Report to hospital | All relevant parties informed | — |
| Follow‑up | — | Inform head of staff on next workday | — | — | — | Follow‑up offered to personnel (unclear if relatives included) | Follow‑up offered to personnel and relatives | Follow‑up dialogue offered to personnel and relatives | — |
| Liability / culpability | — | — | — | — | — | Legal liability clarified | — | — | — |
| Comments | — | Responsibility unclear; no follow‑up procedures for staff or relatives | Delay before Police notified; no follow‑up procedures | Checklist well adapted to risk and response needs; no follow‑up | Police contacted last; Relatives not contacted; Instructions prioritises home‑help service | Emphasises need for early Police alert, but instructions delay this | Notes importance of early alert, but procedures delay Police contact | No written prevention instructions Late Police contact No guidance on relatives or follow‑up | Instructions refer to an inaccessible handbook (broken link) |
